# Supplementary material for: Global depression in breast cancer patients: Systematic review and meta-analysis
Source: PLoS One. 2023 Jul 26;18(7):e0287372. doi: 10.1371/journal.pone.0287372 (PMC10370744; doi:10.1371/journal.pone.0287372)
Supplement: Attachment — Submitted filename: Response to reviewers.docx [file pone.0287372.s003.docx]

Response to reviewers

Editor-in-Chief

**PLOS ONE**

**Art. No**: PONE-D-22-23966

Please find the revised version of our manuscript *“Global Depression in Breast Cancer Patients: systematic review and meta-analysis"*: which we would like to resubmit for publication as a review article in PLOS ONE.

Your comments and those of the reviewers were highly insightful and enabled us to greatly improve the quality of our manuscript. In the following pages there are our point-by-point responses to each of the comments of the reviewers as well as your own comments. Also declaration section revised and completed according to journal guideline.

Revisions in the text are shown using green highlight for additions. We hope that the revisions in the manuscript and our accompanying responses will be sufficient to make our manuscript suitable for publication in PLOS ONE.

We shall look forward to hearing from you at your earliest convenience.

Yours sincerely,

***Corresponding Author:** Ahmad Ghashghaee

**Email:** [ahmad.ghashghaee1996@gmail.com](mailto:ahmad.ghashghaee1996@gmail.com)

| Reviewer Number | Original comments of the reviewer | Reply by the author(s) |
| --- | --- | --- |
| Reviews 1 | Abstracts  1) include data sources; study eligibility criteria, participants, and interventions; study evaluation and synthesis methods; results; limitations; conclusions and implications of key findings;  2) The search was performed more than 6 month ago so I think authors should update their search  3) Keywords: are these keywords are Mesh terms? Word that serves as a keyword, as to the meaning of that condition must be a Mesh term | 1) Since there is word limitation of Abstract, so I cannot add all the materials into this section. However some items has been added.  2) It is impossible to update the research because it is a global systematic review and it will take another 6 month. Also based on evidence, less than two years would be acceptable for a massive systematic reviews like this.  3) Keywords have been changed |
| Reviews 1 | Introduction  The Introduction needs adjustments in order to answer these questions:  - What are the uncertainties and conflicts that underlie the hypotheticals?  - How important is the evidence of studies for the healthy individuals and patients?  - What is the focused clinical question your review will address?  - There are some sentences that are difficult to understand, and the paper needs an English reviewer. Please edit. | This section has been revised. |
| Reviews 1 | Methods  -List and define all variables for which data were sought (e.g., PICOS, funding sources) and any assumptions and simplifications made.  - I suggest not including in the search strategy the outcome`s terms. The search should have more sensitivity than specificity.  -Why authors a priori used a random-effects model for their analysis? Where there any signs to expect significant heterogeneity from the studies included?  .  - it is not necessary to mention all keywords and syntaxes in the method section, authors can refer to main keywords in the methods and present full search strategy and syntaxes in a supplemental file.  - The search was performed more than 6 month ago so I think authors should update their search  - What are these key words? It is appears that they are suitable for just MEDLINE search, so what about other databases? I cannot search SCOPUS with these syntaxes. If the authors use different syntaxes, they should present them, if they don’t at least present keyword in plain form not in a specific database format. | 1) All the variables have been listed  2) Search strategy has been deleted  3) Yes, Since it is a global systematic review and we included various studies for analyzing, random-effect would be the best method for avoiding heterogeneity. |
| Reviews 1 | Discussion  Authors should also acknowledge some serious limitations of the study:  1. significant heterogeneity was encountered perhaps due to various regimens, doses, duration, center settings, populations enrolled etc. calling for cautious interpretation of the results. This is a serious limitation and should be included because it may significantly undermine the validity of results.  2. many of the studies suffer from significant sources of bias and this should be also taken into consideration  3. the effect in many occasions was assessed by very few studies; thus, the evidence to support it is low and should be mentioned. | The Limitation section has been added. |
|  |  |  |
