# Supplementary material for: Global depression in breast cancer patients: Systematic review and meta-analysis
Source: PLoS One. 2023 Jul 26;18(7):e0287372. doi: 10.1371/journal.pone.0287372 (PMC10370744; doi:10.1371/journal.pone.0287372)
Supplement: Attachment — Submitted filename: Response to reviewers.docx [file pone.0287372.s004.docx]

Response to reviewers

Editor-in-Chief

**PLOS ONE**

**Art. No**: PONE-D-22-23966R1

Please find the revised version of our manuscript *“Global Depression in Breast Cancer Patients: systematic review and meta-analysis"*: which we would like to resubmit for publication as a review article in PLOS ONE.

Your comments and those of the reviewers were highly insightful and enabled us to greatly improve the quality of our manuscript. In the following pages there are our point-by-point responses to each of the comments of the reviewers as well as your own comments. Also declaration section revised and completed according to journal guideline.

Revisions in the text are shown using green highlight for additions. We hope that the revisions in the manuscript and our accompanying responses will be sufficient to make our manuscript suitable for publication in PLOS ONE.

We shall look forward to hearing from you at your earliest convenience.

Yours sincerely,

***Corresponding Author:** Ahmad Ghashghaee

**Email:** [ahmad.ghashghaee1996@gmail.com](mailto:ahmad.ghashghaee1996@gmail.com)

| Reviewer Number | Original comments of the reviewer | Reply by the author(s) |
| --- | --- | --- |
| Reviews 2 | Cancer is well-known associated with depression. The association have been discussed in many articles previously. For example, "Pilevarzadeh, M., Amirshahi, M., Afsargharehbagh, R., Rafiemanesh, H., Hashemi, S. M., & Balouchi, A. (2019). Global prevalence of depression among breast cancer patients: a systematic review and meta-analysis. Breast cancer research and treatment, 176, 519-533." and the topic is almost the same with your manuscript. I suggest that your manuscript should include some novel idea about association between breast cancer and depression. In addition, provider supplemental for more detail about your systematic review and meta-analysis.  In addition, for different kinds of depression such as major depressive disorder, dysthymic disorder, and other depressive disorder, combined medications and psychotherapy are the standard treatment and should be mentioned in the introduction and conclusion section. | Firstly, our search period is from 2000-2021 which means we had collected to more years in comparison with mentioned article. Our exclusion and inclusion criteria are different. Also we have result about depression in bearst cancer patients based on Age, stage of treatment and countries and continets which is our novelty.  This issue was not among the main objectives of our study. Our main focus in this article was the prevalence rate in different territories and the relationship between depression and the main variables. |
|  |  |  |
