# Supplementary material for: Global depression in breast cancer patients: Systematic review and meta-analysis
Source: PLoS One. 2023 Jul 26;18(7):e0287372. doi: 10.1371/journal.pone.0287372 (PMC10370744; doi:10.1371/journal.pone.0287372)
Supplement: Attachment — Submitted filename: Response to reviewers.docx [file pone.0287372.s005.docx]

Response to reviewers

Editor-in-Chief

**PLOS ONE**

**Art. No**: PONE-D-22-23966R1

Please find the revised version of our manuscript *“Global Depression in Breast Cancer Patients: systematic review and meta-analysis"*: which we would like to resubmit for publication as a review article in PLOS ONE.

Your comments and those of the reviewers were highly insightful and enabled us to greatly improve the quality of our manuscript. In the following pages there are our point-by-point responses to each of the comments of the reviewers as well as your own comments. Also declaration section revised and completed according to journal guideline.

Revisions in the text are shown using green highlight for additions. We hope that the revisions in the manuscript and our accompanying responses will be sufficient to make our manuscript suitable for publication in PLOS ONE.

We shall look forward to hearing from you at your earliest convenience.

Yours sincerely,

***Corresponding Author:** Ahmad Ghashghaee

**Email:** [ahmad.ghashghaee1996@gmail.com](mailto:ahmad.ghashghaee1996@gmail.com)

| Reviewer Number | Original comments of the reviewer | Reply by the author(s) |
| --- | --- | --- |
| Reviews 2 | 1) The authors have addressed the comments from the reviewers and revised accordingly.  2) Please have English as native language to edit the entire manuscript.  3) Authors should make sure all the fonts and sizes are the same throughout the manuscript. | All requirments have been addressed.  Also a native speaker has revised the language (Author name: Elmira Nosrati Sanjabad) |
|  |  |  |
